# Non-invasive Ventilation and CPAP Failure in Children and Indications for Invasive Ventilation

**DOI:** 10.3389/fped.2020.544921

**Published:** 2020-10-26

**Authors:** Alessandro Amaddeo, Sonia Khirani, Lucie Griffon, Theo Teng, Agathe Lanzeray, Brigitte Fauroux

**Affiliations:** ^1^Pediatric Noninvasive Ventilation and Sleep Unit, AP-HP, Hôpital Necker-Enfants malades, Paris, France; ^2^Université de Paris, VIFASOM, Paris, France; ^3^ASV Sante, Gennevilliers, France

**Keywords:** CPAP (continuous positive air pressure), ENT surgeries, sleep disordered breathing (obstructive/central sleep apnea), NIV failure, educational therapeutic assistance

## Abstract

Non-invasive ventilation (NIV) and continuous positive airway pressure (CPAP) are effective treatments for children with severe sleep disordered breathing (SBD). However, some patients may present too severe SDB that do not respond to NIV/CPAP or insufficient compliance to treatment. A careful revaluation of the interface and of ventilator settings should be performed before considering alternative treatments. In patients with obstructive sleep apnea (OSA), alternatives to CPAP/NIV rely on the underlying disease. Ear-nose-throat (ENT) surgery such as adeno-tonsillectomy (AT), turbinectomy or supraglottoplasty represent an effective treatment in selected patients before starting CPAP/NIV and should be reconsidered in case of CPAP failure. Rapid maxillary expansion (RME) is restricted to children with OSA and a narrow palate who have little adenotonsillar tissue, or for those with residual OSA after AT. Weight loss is the first line therapy for obese children with OSA before starting CPAP and should remain a priority in the long-term. Selected patients may benefit from maxillo-facial surgery such as mandibular distraction osteogenesis (MDO) or from neurosurgery procedures like fronto-facial monobloc advancement. Nasopharyngeal airway (NPA) or high flow nasal cannula (HFNC) may constitute efficient alternatives to CPAP in selected patients. Hypoglossal nerve stimulation has been proposed in children with Down syndrome not tolerant to CPAP. Ultimately, tracheostomy represents the unique alternative in case of failure of all the above-mentioned treatments. All these treatments require a multidisciplinary approach with a personalized treatment tailored on the different diseases and sites of obstruction. In patients with neuromuscular, neurological or lung disorders, non-invasive management in case of NIV failure is more challenging. Diaphragmatic pacing has been proposed for some patients with central congenital hypoventilation syndrome (CCHS) or neurological disorders, however its experience in children is limited. Finally, invasive ventilation via tracheotomy represents again the ultimate alternative for children with severe disease and little or no ventilatory autonomy. However, ethical considerations weighting the efficacy against the burden of this treatment should be discussed before choosing this last option.

## Introduction

Long term non-invasive ventilation (NIV) and continuous positive pressure (CPAP) are increasingly used in children with sleep disordered breathing (SDB) (1–4). The choice of the respiratory support (NIV or CPAP), relies on the underlying physiopathological mechanisms. CPAP is the treatment of choice in severe upper airway obstruction as in obstructive sleep apnea (OSA) (3, 5, 6) or in some cases of lower upper airway involvement as in patients with tracheo or bronchomalacia or bronchopulmonary dysplasia (BPD) (7). NIV represents the first line treatment for chronic respiratory failure associated with neuromuscular disorders (3, 8, 9), central nervous system abnormalities (10, 11), lung diseases (12, 13), chest wall deformities or obesity and hypoventilation (3, 5, 14). Independently from the underlying disease, the aim of the treatment is to normalize overnight and daytime gas exchange and sleep efficiency, improve neurocognitive outcomes, and decrease morbidity and mortality. These benefits may be achieved if NIV or CPAP are able to efficiently counteract the pathological abnormalities responsible of SDB and if treatment adherence is sufficient. Therefore, NIV or CPAP may fail because of a too severe disease or because the child refuses or do not tolerate the NIV/CPAP for a sufficient amount of time.

Alternatives to a non-invasive ventilatory support vary according to the underlying disease severity, the patient's behavioral and cognitive status, the family support and the expected benefit of NIV/CPAP treatment. Treatment options change with child's age and the need for NIV/CPAP requires a constant evaluation. From a practical point of view, treatment failure and therapeutic options must be evaluated in the light of the underlying pathological mechanism and the expected benefit. This review analyzes the current alternative therapeutic options to CPAP and NIV in children with OSA and in children with nocturnal hypoventilation due to neuromuscular, neurological, thoracic, and lung disorders.

## Obstructive Sleep Apnea (OSA)

OSA is defined as the “recurrent partial or complete upper airway obstruction (hypopneas, obstructive or mixed apneas) with disruption of normal oxygenation, ventilation and sleep pattern (15).” The prevalence of OSA in children varies widely from 0.1 to 13% (16). OSA is classically associated with tonsillar hypertrophy in otherwise healthy children, and most of the literature data concerns this population. These children normally have mild to moderate OSA that resolves after adeno-tonsillectomy (AT), and do not require long term CPAP (6, 17, 18). OSA is more common and more severe in children with associated conditions such as congenital craniofacial malformations [e.g., Pierre Robin syndrome (19), complex craniofacial abnormalities (20–22), syndromic craniostenosis (23–25)], metabolic or endocrinology disorders [Prader Willi syndrome (20, 26, 27), storage diseases (21, 28)] or genetic conditions [Down syndrome (22, 29, 30)]. The age of these children with “complex OSA” ranges from newborns to young adults. In children with complex OSA the airway obstruction is most often multifactorial and multilevel requiring an objective assessment and treatment of the different abnormalities that contribute to OSA (31). Moreover, they are also more likely to have persistent OSA after upper airway surgery and represent the large majority of children treated with CPAP (1–3, 5). No data are available in the pediatric population, however the expected benefits of CPAP on nocturnal gas exchange, sleep disruption and cardiovascular outcomes are related to CPAP compliance, with a probable positive dose–effect relationship (32). CPAP should ideally be used during the total physiological sleep time, which may exceed 12 h in infants (e.g., Pierre Robin, Treacher-Collins syndrome). No minimal CPAP/NIV utilization has been validated in children. Some authors propose a minimal use of more than 4 h per night for at least 70% of the nights over a 30 days period (33) while others propose more of 50% of the total sleep time (34). In our center, we recommend an utilization of at least 6 h per night for more than 80% of nights (35).

### Checking of Equipment and Educational Support

**Figure 1:**
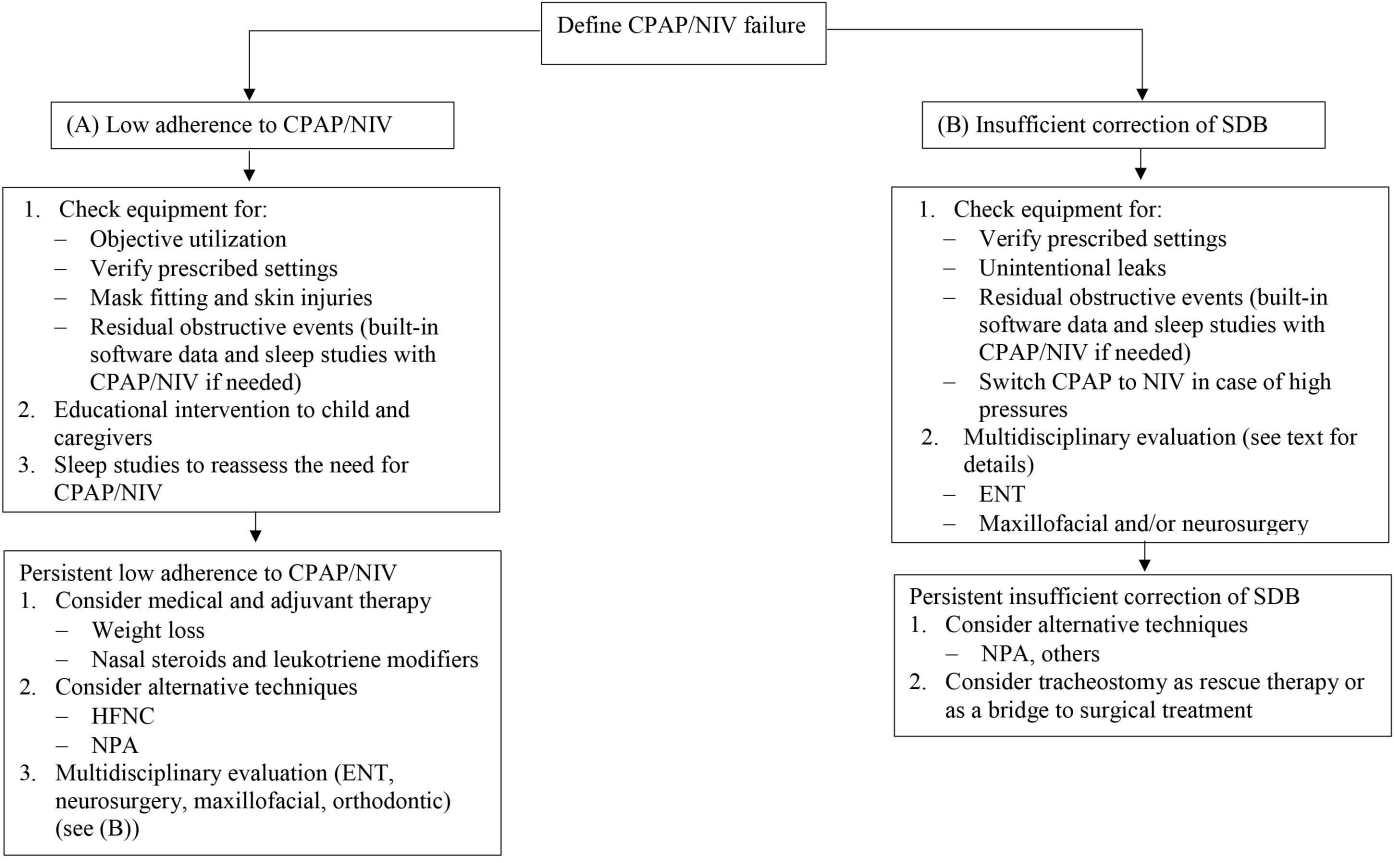
Proposed algorithm for CPAP and NIV failure in OSA. CPAP, continuous positive airway pressure; NIV, non-invasive ventilation; OSA, obstructive sleep apnea; HFNC, high flow nasal cannula; NPA, nasopharyngeal airway; ENT, ear nose and throat; SDB, sleep disordered breathing.

Objective assessment of CPAP/NIV utilization is mandatory. First of all, in case low adherence, the first step is to check the equipment. Figure 1 shows a proposed algorithm for alternatives to CPAP/NIV in cases of OSA. As almost all CPAP and NIV devices have built-in software, the first step is to check this data in order to identify possible pitfalls. Device settings must correspond to the prescribed settings. As most of devices have been designed for adult patients, manufactures indicate a minimal weight for correct detection of patients flow. In case of infants, this may underestimate the real use of the device (33, 36). The detailed analysis of the overnight pattern may identify troubleshoot issues like frequent nighttime awakenings or feeding routine in infants (34). Careful evaluation of unintentional leaks should be performed since unintentional leaks may cause conjunctival irritation or excessive mouth dryness. The proper mask fitting should be checked in order to rapidly correct skin injury which is common in infants and children with craniofacial malformations.

The combined analysis of in-built software data and nocturnal pulse oximetry or a sleep study with CPAP/NIV may identify residual respiratory events that require settings changes (35, 37, 38). Autotitrating CPAP may be used as a tool to titrate CPAP level in older children (39). Switch from CPAP to NIV must be considered when high pressures are needed to correct residual obstructive events or if nocturnal hypoventilation persists despite optimal CPAP.

The patient's and caregiver's motivation and support should be assessed if the compliance remains low after checking of all technical issues. Most studies reported a suboptimal compliance with CPAP uses ranging from 4.7 to 5.3 h/night despite a behavioral program and a close follow-up (40, 41). Supportive interventions, educational interventions and behavioral therapy have proven their efficacy to improve CPAP compliance in adult patients (42). To our knowledge, only one study evaluated the effect of educational interventions on CPAP compliance in children, and showed that the benefit of education intervention was maintained over time in 3 out of 4 preschool children (43). Importantly, especially for children, it is crucial to choose age- and developmental-adjusted interventions that best match individual patient needs in order to reach the most successful and cost-effective therapy (35). Finally, in those children who have a disease that may improve with age and whose adherence decreases, a sleep study is recommended in order to assess the possibility of CPAP or NIV weaning.

### Management of CPAP Failure in OSA

OSA may change with child's growth, with some children improving spontaneously while other deteriorate, justifying a continuous multidisciplinary assessment (44). Despite a high rate of residual OSA after AT, repeated or alternative upper airway surgery in children with craniofacial abnormalities or neurological disorders may cure or improve OSA (45–49). Surgical procedures other than AT may be indicated in children with “complex OSA.” In this selected population drug induced sleep endoscopy (DISE) allows the identification of the actual site(s) of obstruction, allowing an optimal individualized treatment (50). Based on the type and site(s) of obstruction, common alternative approaches are turbinectomy, lingual tonsillectomy, supraglottoplasty, uvulopalatopharyngoplasty (UPPP), mandibular distraction osteogenesis (MDO), tongue base procedures, and tongue-lip adhesion. Inferior turbinate hypertrophy (ITH) is a common cause of nasal obstruction in children, especially in those with craniofacial abnormalities. The aim of turbinectomy is to reduce bony turbinate and erectile submucosal tissue while preserving warming and humidification of inspired air. Turbinectomy can be performed at any age and may be associated with adenoidectomy in selected cases depending on patient's anatomical characteristics (51). UPPP is a technique widely used for the management of OSA in adults but not in children. It has mainly been used in children at high risk for persistent upper airway obstruction after AT alone, including children with Down syndrome and neurological disorders (52–54). Potential complications include nasopharyngeal stenosis, palatal incompetence, and speech difficulties. However, the small sample size of the studies, the absence of control groups, and paucity of validated outcome measures preclude any conclusion on the usefulness of this procedure in a broader pediatric population. In children with obesity and Down syndrome, obstruction at the base of tongue is increasingly recognized as a cause of persistent OSA after AT (55–60). Endoscopic-assisted coblation lingual tonsillectomy is effective to treat tongue base collapse in children with OSA (61). Newer techniques like trans oral robotic surgery (TORS) allow an accurate view of lingual base and a better and more efficient dissection of tissues. This may improve the efficacy of the procedure as shown in a retrospective case series of syndromic and non-syndromic patients with residual OSA after AT or with (CPAP) failure (62).

Rapid maxillary expansion (RME) is an orthodontic treatment that widens the palate and nasal passage, thereby increasing airway caliber and reducing nocturnal upper airway obstruction. The technique can only be used prior to midline fusion of the maxilla, which generally occurs shortly prior to puberty. RME is proposed after the 4th year of life, when the second deciduous molars of the upper jaw have erupted, and the device is usually removed after a period of 12 months (63). RME is restricted to children with OSA and a narrow palate (crossbite) who have little adenotonsillar tissue, or for those with residual OSA after AT. Orthopedic mandibular advancement (OMA) aims to correct dental and skeletal retrognathia by re-directing the mandible into a more forward and downward position. This aims at increasing the opening of the oropharyngeal airway during wake and sleep. Despite its large use in adults as an alternative to CPAP (64, 65) only few studies analyzed the efficacy of this treatment in children. In young adolescents, OMA has been shown to be an effective technique to correct moderate OSA with a mean apnea-hypopnea index (AHI) reduction of 5 events/h (66).

MDO consists in the application of internal or external distraction devices that are placed on each mandibular branch. MDO has been used with success to treat severe OSA due to mandibular hypoplasia, particularly in infants with congenital mandibular hypoplasia such as Pierre Robin sequence or Treacher Collins (67–69). Successful treatment of airway obstruction (defined as either tracheotomy avoidance or decannulation, no need for CPAP, or significant improvement or absence of OSA symptoms) is reported in almost 90% of children by a recent meta-analysis (70). However, rate of post-operative complications is high, with more than 20% of patients having osteomyelitis, epidural abscess, open bite deformity, nerve injuries, or hypertrophic scarring. Little information is available concerning the long term efficacy of MDO performed early in life (71). These data suggest that MDO may be an option for severe OSA in selected patients with mandibular hypoplasia related to congenital craniofacial defects, but the high rate of complications and the possible reoccurrence of OSA limits it use. Other surgical techniques like frontofacial monobloc advancement, or Lefort type 2 or 3 are reserved to congenital facial bone deformities such as faciocraniodysostosis (72–75).

Tracheotomy is an invasive technique that represents the ultimate rescue therapy for children with severe OSA for whom no other therapeutic options are available. On the other hand, in many cases tracheostomy represents a safe and easy to manage therapy. Moreover, practice often depends on local training habits and on the expertise of the different centers, with some team preferring tracheostomy for failure of conservative methods (76). Although tracheotomy is an effective treatment for severe OSA, it may be associated with complications, such scaring, granulous tissue, bleeding, tracheoesophageal fistula, accidental decannulation, tube occlusion or even death, in around 43–77% of children (77, 78). However, some of these complications may be reduced by choosing between the different surgical techniques, or by having particular care in the choice of cannula quality and positioning. Finally, before performing the tracheostomy a complete airway endoscopy is recommended in patients with complex OSA since many of them may present with associated tracheal bronchial malformations that could reduce the efficacy of tracheostomy. Therefore, even if life–saving in some cases, the decision for a tracheotomy should be carefully discussed with a multidisciplinary team, the patient and the parents. It is also important to note that tracheostomy in this setting may be considered as a bridge therapy to lesser invasive methods since most of these children and infants may have a progressive spontaneous improvement of airway obstruction with age or a decrease of OSA severity after surgical treatment allowing a post-operative decannulation (79–81).

### Other Therapies

Hypoglossal nerve stimulation is a technique that consists in an implantable device that delivers electrical impulses to tongue protrusor muscles during inspiration aiming at reducing the collapsibility of the upper airway. Hypoglossal nerve stimulation has been shown to be effective in selected adults with OSA and no obesity or circumferential velopharynx collapse (82, 83). This technique has been proposed in some children and adolescents with Down syndrome who had persistent OSA after ENT surgery and who were not adherent to CPAP treatment with a significant improvement in the AHI and quality of life (84, 85), with no surgical complications. However, information on long term follow up is lacking.

Nasopharyngeal airway (NPA) refers to the placement of a modified endotracheal tube in the hypopharynx. In infants with Pierre Robin syndrome or other congenital abnormalities of the upper airway, NPA has shown to be effective and well-tolerated, even if 10–20% of children still require tracheostomy (86–88). The technique is limited to some specialized centers and complications such as displacement of the tube and nostril stenosis have been reported (89).

High flow nasal cannula (HFNC) is a non-invasive ventilatory device that is increasingly used for the treatment of acute and chronic respiratory failure in adults and children (90, 91). HFNC consists of the delivery of high flowing heated and humidified air through the nose, with a fraction of oxygen (FiO_2_) that may be set from 21% to nearly 100%. The nasal cannulas used with HFNC are less invasive and more comfortable than nasal masks or nasal prongs used for CPAP therapy. A few case series have reported the use of HFNC in children with OSA (91–93). Our group recently reported the efficacy of HFNC as a rescue therapy for OSA in children and infants non-compliant to CPAP (94). Also, the use of HFNC interface with a modified circuit connected to a standard ventilator has been reported in infants and children not tolerant to CPAP (95).

Some adjuvant therapies may be indicated for selected patients. Weight loss should be the first line therapy for obese children with OSA (6, 15). However, the presumption that weight loss is beneficial in obese children with OSA is based primarily upon evidence from adults. This may be explained by the difficulty to achieve a sufficient weight loss in the large majority of obese children. The few studies that have evaluated the effect of weight loss on OSA in obese children suggested that OSA is likely to improve if a sufficient weight loss is achieved. Verhulst et al. reported that weight loss was able to improve OSA in 62% of obese teenagers attending a residential treatment center (96). Similar results were reported by Siegfried and colleagues in a similar setting of obese adolescents admitted to a rehabilitation center (97). However, it has to be noted that weight loss achieved in a rehabilitation center may not be extended to home. Moreover, patients with morbid obesity due to some genetical disorders like Prader Willi syndrome or rapid-onset obesity with hypothalamic dysregulation, hypoventilation, and autonomic dysregulation (ROHHAD) weight loss programs are frequently ineffective. For adolescents with morbid obesity and OSA and/or other obesity-associated morbidities, weight loss surgery may be an option (98, 99).

Intranasal corticosteroids or leukotriene modifier therapy have been shown to improve children with mild surgery-naïve OSA or mild residual OSA after AT. This treatment may decrease nasal inflammation and improve nasal obstruction especially in those children with chronic nasal obstruction due to chronic allergic rhinitis or who had difficulties in tolerating CPAP/NIV nasal interfaces.

Positional therapy may be considered when a school-aged child, adolescent, or older teenager presents obstructive respiratory events, occurring exclusively or predominantly in the supine position as compared to the other positions. Several tools such as pillows and belts are commercially available. Positional therapy has not been well-evaluated in children, and a sleep study should be performed to verify its efficacy case per case.

### Management of NIV Failure

Hypoventilation, defined by hypercapnia and hypoxemia, is the consequence of an imbalance between respiratory load and the ability of the respiratory muscles to generate an adequate alveolar ventilation (100). Some patients may have clinical unapparent hypoventilation without hypoxemia but others present with associated oxygen desaturations. In these latter the administration of oxygen alone may lead to a worsening of hypoventilation. Since in most of the cases the underlying disorders relies on respiratory muscles weakness or central drive failure, NIV and not CPAP is the treatment of choice to restore normal nocturnal gas exchange. Most of the patients have progressive chronic diseases such as neuromuscular or neurological disorders with little or no improvement over time. This means that, in the majority of cases, NIV represents a long-term treatment and that persistent treatment adherence is of paramount importance. In most of these patients, and in particular in those with neuromuscular disorders, NIV is initially used during the night since nocturnal hypoventilation is the predominant abnormality in the first stages of the disease. In case of low or inadequate adherence to treatment the first step is to check equipment for common pitfalls, as already described for OSA patients. Particular attention should be given to the detection of patient-ventilator asynchronies and unintentional leaks (often mouth leaks), since these represent the most common residual respiratory events in children treated with NIV (101). Then, side effects such as skin injury due to pressure sores, eye irritation due to unintentional air leaks, facial deformity (102) or aerophagia and gastric distension should be looked for and managed promptly. As for patients with OSA, educational interventions and behavioral support should be offered to patients and their families.

Patients with congenital central hypoventilation syndrome (CCHS) and those with spinal cord injury and elective diaphragm palsy may be eligible for diaphragm pacing (DP). DP consists on an implantable device that sends electrical impulses to the diaphragm via the phrenic nerve. Successful decannulation and management of SDB in pediatric CCHS patients have been reported, even though treatment efficacy should be carefully assessed because this approach may not be sufficient or cause obstructive respiratory events (103, 104).

Finally, when respiratory insufficiency progress, especially in children with advanced neuromuscular disease, NIV may fail to guarantee efficient correction of nocturnal and daytime gas exchange. Some groups reported successful experience with NIV in totally ventilator-dependent children with neuromuscular disease (105), while others consider that a minimal time per day of spontaneous breathing is needed in order to balance medical benefit and quality of life of the child and his family (106). In this case invasive ventilation (IV), may represent an alternative to NIV although it represents an option which has to be prepared and discussed thoroughly with the child and his parents (107). In children with a severe underlying disease with a rapidly fatal outcome, developmental delay or severe physical or cognitive disabilities tracheostomy may be even more controversial since the transition from NIV to IV may be associated with a too high burden with regard to the improvement in quality of life of the child and his family (108).

## Conclusion

CPAP and NIV are widely used to treat SDB in infants and children. However, their efficacy depends on treatment adherence and underlying diseases severity. Checking of equipment and patient's and caregiver's education are of paramount importance to achieve an optimal CPAP/NIV use. For patients with OSA and CPAP failure, a multidisciplinary approach is recommended in order to choose the most appropriate therapeutic option. In patients with nocturnal hypoventilation due to neuromuscular or neurological conditions, the range of alternative therapies is limited. Tracheostomy with or without invasive ventilation represents an option that need to be discussed with the patients and families, and ethical considerations weighting the efficacy against the burden of this treatment should be discussed.

## Author Contributions

AA is the main author of the manuscript. BF, SK, LG, TT, and AL contributed to the conception of the manuscript. All authors approved the final version of the manuscript.

## Conflict of Interest

SK is employed by the company ASV Sante but does not have any commercial of financial conflict of interest with the content of this review. The remaining authors declare that the research was conducted in the absence of any commercial or financial relationships that could be construed as a potential conflict of interest.

## References

[1] Castro-Codesal ML Dehaan K Bedi PK Bendiak GN Schmalz L Katz SL. Longitudinal changes in clinical characteristics and outcomes for children using long-term non-invasive ventilation. PLoS ONE. (2018) 13:e0192111. 10.1371/journal.pone.0192111 29381756 PMC5790245

[2] Pavone M Verrillo E Caldarelli V Ullmann N Cutrera R. Non-invasive positive pressure ventilation in children. Early Hum. Dev. (2013) 89(Suppl. 3):S25–31. 10.1016/j.earlhumdev.2013.07.019 23958409

[3] Amaddeo A Moreau J Frapin A Khirani S Felix O Fernandez-Bolanos M. Long term continuous positive airway pressure (CPAP) and noninvasive ventilation (NIV) in children: initiation criteria in real life: long Term CPAP and NIV in Children. Pediatr. Pulmonol. (2016) 51:968–74. 10.1002/ppul.23416 27111113

[4] Amin R Sayal P Syed F Chaves A Moraes TJ MacLusky I. Pediatric long-term home mechanical ventilation: twenty years of follow-up from one Canadian center. Pediatr. Pulmonol. (2014) 49:816–24. 10.1002/ppul.22868 24000198

[5] Girbal IC Gonçalves C Nunes T Ferreira R Pereira L Saianda A. Non-invasive ventilation in complex obstructive sleep apnea – A 15-year experience of a pediatric tertiary center. Rev. Port. Pneumol. (2014) 20:146–51. 10.1016/j.rppnen.2014.05.001 24525398

[6] Kaditis AG Alonso Alvarez ML Boudewyns A Alexopoulos EI Ersu R Joosten K. Obstructive sleep disordered breathing in 2- to 18-year-old children: diagnosis and management. Eur. Respir. J. (2016) 47:69–94. 10.1183/13993003.00385-2015 26541535

[7] Khirani S Ramirez A Aloui S Leboulanger N Picard A Fauroux B Continuous positive airway pressure titration in infants with severe upper airway obstruction or bronchopulmonary dysplasia. Crit. Care. (2013) 17:R167 10.1186/cc12846 23889768 PMC4056687

[8] Finkel RS Mercuri E Meyer OH Simonds AK Schroth MK Graham RJ Diagnosis and management of spinal muscular atrophy: part 2: pulmonary and acute care; medications, supplements and immunizations; other organ systems; and ethics. Neuromuscul. Disord. (2018) 28:197–207. 10.1016/j.nmd.2017.11.004 29305137

[9] Hull J Aniapravan R Chan E Chatwin M Forton J Gallagher J British Thoracic Society guideline for respiratory management of children with neuromuscular weakness. Thorax. (2012) 67(Suppl. 1):i1–40. 10.1136/thoraxjnl-2012-201964 22730428

[10] Grychtol R Chan EY. Use of non-invasive ventilation in cerebral palsy. Arch. Dis. Child. (2018) 103: 1170–7. 10.1136/archdischild-2017-313959 29886412

[11] Weese-Mayer DE Berry-Kravis EM Ceccherini I Keens TG Loghmanee DA Trang H. An official ATS clinical policy statement: congenital central hypoventilation syndrome. Am. J. Respir. Crit. Care Med. (2010) 181:626–44. 10.1164/rccm.200807-1069ST 20208042

[12] Fauroux B Burgel P-R Boelle P-Y Cracowski C Murris-Espin M Nove-Josserand R. Practice of noninvasive ventilation for cystic fibrosis: a nationwide survey in France. Respir. Care. (2008) 53:1482–9. 18957151

[13] Moran F Bradley JM Piper AJ. Non-invasive ventilation for cystic fibrosis. Cochrane Database Syst. Rev. (2017) 2:CD002769. 10.1002/14651858.CD002769.pub5 28218802 PMC6464053

[14] Edwards EA Hsiao K Nixon GM. Paediatric home ventilatory support: the Auckland experience. J. Paediatr. Child Health. (2005) 41:652–8. 10.1111/j.1440-1754.2005.00753.x 16398869

[15] Marcus CL Brooks LJ Ward SD Draper KA Gozal D Halbower AC Diagnosis and management of childhood obstructive sleep apnea syndrome. Pediatrics. (2012) 130:e714–55. 10.1542/peds.2012-1672 22926176

[16] Bixler EO Vgontzas AN Lin H-M Liao D Calhoun S Vela-Bueno A Sleep disordered breathing in children in a general population sample: prevalence and risk factors. Sleep. (2009) 32:731–6. 10.1093/sleep/32.6.731 19544748 PMC2690559

[17] Bhattacharjee R Kheirandish-Gozal L Spruyt K Mitchell RB Promchiarak J Simakajornboon N. Adenotonsillectomy outcomes in treatment of obstructive sleep apnea in children: a multicenter retrospective study. Am. J. Respir. Crit. Care Med. (2010) 182:676–83. 10.1164/rccm.200912-1930OC 20448096

[18] Lee C-H Hsu W-C Chang W-H Lin M-T Kang K-T. Polysomnographic findings after adenotonsillectomy for obstructive sleep apnoea in obese and non-obese children: a systematic review and meta-analysis. Clin. Otolaryngol. (2016) 41:498–510. 10.1111/coa.12549 26436726

[19] Robin P La chute de la base de la langue considérée comme une nouvelle cause de gène dans la respiration naso-pharyngienne. Bull. Acad. Natl. Med. (1923) 89:37–41.

[20] Cohen M Hamilton J Narang I. Clinically important age-related differences in sleep related disordered breathing in infants and children with Prader-Willi syndrome. PLoS ONE. (2014) 9:e101012. 10.1371/journal.pone.0101012 24979549 PMC4076199

[21] Lin H-Y Chen M-R Lin C-C Chen C-P Lin D-S Chuang C-K. Polysomnographic characteristics in patients with mucopolysaccharidoses. Pediatr. Pulmonol. (2010) 45:1205–12. 10.1002/ppul.21309 20717914

[22] Fan Z Ahn M Roth HL Li L Vaughn BV. Sleep apnea and hypoventilation in patients with down syndrome: analysis of 144 polysomnogram studies. Children. (2017) 4:55. 10.3390/children4070055 28665356 PMC5532547

[23] Moraleda-Cibrián M Edwards SP Kasten SJ Buchman SR Berger M O'Brien LM. Obstructive sleep apnea pretreatment and posttreatment in symptomatic children with congenital craniofacial malformations. J. Clin. Sleep Med. (2015) 11:37–43. 10.5664/jcsm.4360 25515281 PMC4265656

[24] Alsaadi MM Iqbal SM Elgamal EA Salih MA Gozal D. Sleep-disordered breathing in children with craniosynostosis. Sleep Breath. (2013) 17:389–93. 10.1007/s11325-012-0706-2 22535197

[25] Al-Saleh S Riekstins A Forrest CR Philips JH Gibbons J Narang I. Sleep-related disordered breathing in children with syndromic craniosynostosis. J. Craniomaxillofac. Surg. (2011) 39:153–7. 10.1016/j.jcms.2010.04.011 20627744

[26] Lin H-Y Lin S-P Lin C-C Tsai L-P Chen M-R Chuang C-K. Polysomnographic characteristics in patients with Prader-Willi syndrome. Pediatr. Pulmonol. (2007) 42:881–7. 10.1002/ppul.20673 17722117

[27] Pavone M Caldarelli V Khirani S Colella M Ramirez A Aubertin G. Sleep disordered breathing in patients with Prader-Willi syndrome: a multicenter study. Pediatr. Pulmonol. (2015) 50:1354–9. 10.1002/ppul.23177 25851435

[28] Moreau J Brassier A Amaddeo A Neven B Caillaud C Chabli A. Obstructive sleep apnea syndrome after hematopoietic stem cell transplantation in children with mucopolysaccharidosis type I. Mol. Genet. Metab. (2015) 116:275–80. 10.1016/j.ymgme.2015.10.004 26602600

[29] de Miguel-Díez J Villa-Asensi JR Alvarez-Sala JL. Prevalence of sleep-disordered breathing in children with down syndrome: polygraphic findings in 108 children. Sleep. (2003) 26:1006–9. 10.1093/sleep/26.8.1006 14746382

[30] Alexander M Petri H Ding Y Wandel C Khwaja O Foskett N. Morbidity and medication in a large population of individuals with down syndrome compared to the general population. Dev. Med. Child. Neurol. (2016) 58:246–54. 10.1111/dmcn.12868 26282180

[31] Kaditis AG Alonso Alvarez ML Boudewyns A Abel F Alexopoulos EI Ersu R ERS statement on obstructive sleep disordered breathing in 1- to 23-month-old children. Eur. Respir. J. (2017) 50:1700985 10.1183/13993003.00985-2017 29217599

[32] Barbé F Durán-Cantolla J Capote F de la Peña M Chiner E Masa JF. Long-term effect of continuous positive airway pressure in hypertensive patients with sleep apnea. Am. J. Respir. Crit. Care Med. (2010) 181:718–26. 10.1164/rccm.200901-0050OC 20007932

[33] Machaalani R Evans CA Waters KA. Objective adherence to positive airway pressure therapy in an Australian paediatric cohort. Sleep Breath. (2016) 20:1327–36. 10.1007/s11325-016-1400-6 27591801

[34] Perrem L Mehta K Syed F Baker A Amin R. How to use noninvasive positive airway pressure device data reports to guide clinical care. Pediatr. Pulmonol. (2020) 55:58–67. 10.1002/ppul.24555 31671252

[35] Amaddeo A Frapin A Touil S Khirani S Griffon L Fauroux B. Outpatient initiation of long-term continuous positive airway pressure in children. Pediatr. Pulmonol. (2018) 53:1422–8. 10.1002/ppul.24138 30070059

[36] Ramirez A Khirani S Aloui S Delord V Borel J-C Pépin J-L. Continuous positive airway pressure and noninvasive ventilation adherence in children. Sleep Med. (2013) 14:1290–4. 10.1016/j.sleep.2013.06.020 24157098

[37] Amaddeo A Caldarelli V Fernandez-Bolanos M Moreau J Ramirez A Khirani S. Polygraphic respiratory events during sleep in children treated with home continuous positive airway pressure: description and clinical consequences. Sleep Med. (2015) 16:107–12. 10.1016/j.sleep.2014.07.030 25541022

[38] Khirani S Delord V Olmo Arroyo J De Sanctis L Frapin A Amaddeo A. Can the analysis of built-in software of CPAP devices replace polygraphy in children? Sleep Med. (2017) 37:46–53. 10.1016/j.sleep.2017.05.019 28899539

[39] Mihai R Vandeleur M Pecoraro S Davey MJ Nixon GM. Autotitrating CPAP as a tool for CPAP initiation for children. J. Clin. Sleep Med. (2017) 13:713–19. 10.5664/jcsm.6590 28356178 PMC5406961

[40] O'Donnell AR Bjornson CL Bohn SG Kirk VG. Compliance rates in children using noninvasive continuous positive airway pressure. Sleep. (2006) 29:651–8. 10.1093/sleep/29.5.651 16774155

[41] Marcus CL. Adherence to and effectiveness of positive airway pressure therapy in children with obstructive sleep apnea. Pediatrics. (2006) 117:e442–51. 10.1542/peds.2005-1634 16510622

[42] Wozniak DR Lasserson TJ Smith I Educational, supportive and behavioural interventions to improve usage of continuous positive airway pressure machines in adults with obstructive sleep apnoea. Cochrane Database Syst. Rev. (2014) 8:CD007736 10.1002/14651858.CD007736.pub2 24399660

[43] Slifer KJ Kruglak D Benore E Bellipanni K Falk L Halbower AC. Behavioral training for increasing preschool children's adherence with positive airway pressure: a preliminary study. Behav. Sleep Med. (2007) 5:147–75. 10.1080/15402000701190671 17441784

[44] Mastouri M Amaddeo A Griffon L Frapin A Touil S Ramirez A Weaning from long term continuous positive airway pressure or noninvasive ventilation in children. Pediatr. Pulmonol. (2017) 52:1349–54. 10.1002/ppul.23767 28714612

[45] Lefaivre JF Cohen SR Burstein FD Simms C Scott PH Montgomery GL. Down syndrome: identification and surgical management of obstructive sleep apnea. Plast. Reconstr. Surg. (1997) 99:629–37. 10.1097/00006534-199703000-00004 9047180

[46] Afsharpaiman S Sillence DO Sheikhvatan M Ault JE Waters K. Respiratory events and obstructive sleep apnea in children with achondroplasia: investigation and treatment outcomes. Sleep Breath. (2011) 15:755–61. 10.1007/s11325-010-0432-6 21225355

[47] Amonoo-Kuofi K Phillips SP Randhawa PS Lane R Wyatt ME Leighton SEJ. Adenotonsillectomy for sleep-disordered breathing in children with syndromic craniosynostosis. J. Craniofac. Surg. (2009) 20:1978–80. 10.1097/SCS.0b013e3181bd2c9a 19881386

[48] Tenconi R Khirani S Amaddeo A Michot C Baujat G Couloigner V. Sleep-disordered breathing and its management in children with achondroplasia. Am. J. Med. Genet. A. (2017) 173:868–78. 10.1002/ajmg.a.38130 28239978

[49] Dudoignon B Amaddeo A Frapin A Thierry B de Sanctis L Arroyo JO. Obstructive sleep apnea in down syndrome: benefits of surgery and noninvasive respiratory support. Am. J. Med. Genet. A. (2017) 173:2074–80. 10.1002/ajmg.a.38283 28544488

[50] Boudewyns A Saldien V Van de Heyning P Verhulst S. Drug-induced sedation endoscopy in surgically naïve infants and children with obstructive sleep apnea: impact on treatment decision and outcome. Sleep Breath. (2017) 22:503–10. 10.1007/s11325-017-1581-7 29081031

[51] Komshian SR Cohen MB Brook C Levi JR. Inferior turbinate hypertrophy: a review of the evolution of management in children. Am. J. Rhinol. Allergy. (2019) 33:212–9. 10.1177/1945892418815351 30554518

[52] Strome M. Obstructive sleep apnea in down syndrome children: a surgical approach. Laryngoscope. (1986) 96:1340–2. 10.1288/00005537-198612000-00004 2946913

[53] Kosko JR Derkay CS. Uvulopalatopharyngoplasty: treatment of obstructive sleep apnea in neurologically impaired pediatric patients. Int. J. Pediatr. Otorhinolaryngol. (1995) 32:241–6. 10.1016/0165-5876(95)01178-E 7665271

[54] Kerschner JE Lynch JB Kleiner H Flanary VA Rice TB. Uvulopalatopharyngoplasty with tonsillectomy and adenoidectomy as a treatment for obstructive sleep apnea in neurologically impaired children. Int. J. Pediatr. Otorhinolaryngol. (2002) 62:229–35. 10.1016/S0165-5876(01)00623-1 11852126

[55] Truong MT Woo VG Koltai PJ. Sleep endoscopy as a diagnostic tool in pediatric obstructive sleep apnea. Int. J. Pediatr. Otorhinolaryngol. (2012) 76:722–7. 10.1016/j.ijporl.2012.02.028 22421163

[56] Durr ML Meyer AK Kezirian EJ Rosbe KW. Drug-induced sleep endoscopy in persistent pediatric sleep-disordered breathing after adenotonsillectomy. Arch. Otolaryngol. Head Neck Surg. (2012) 138:638–43. 10.1001/archoto.2012.1067 22801887

[57] Shott SR Donnelly LF. Cine magnetic resonance imaging: evaluation of persistent airway obstruction after tonsil and adenoidectomy in children with down syndrome. Laryngoscope. (2004) 114:1724–9. 10.1097/00005537-200410000-00009 15454761

[58] Donnelly LF Shott SR LaRose CR Chini BA Amin RS. Causes of persistent obstructive sleep apnea despite previous tonsillectomy and adenoidectomy in children with down syndrome as depicted on static and dynamic cine MRI. AJR Am. J. Roentgenol. (2004) 183:175–81. 10.2214/ajr.183.1.1830175 15208134

[59] Maris M Verhulst S Saldien V Van de Heyning P Wojciechowski M Boudewyns A. Drug-induced sedation endoscopy in surgically naive children with down syndrome and obstructive sleep apnea. Sleep Med. (2016) 24:63–70. 10.1016/j.sleep.2016.06.018 27810188

[60] Prosser JD Shott SR Rodriguez O Simakajornboon N Meinzen-Derr J Ishman SL. Polysomnographic outcomes following lingual tonsillectomy for persistent obstructive sleep apnea in down syndrome. Laryngoscope. (2017) 127:520–4. 10.1002/lary.26202 27515709

[61] Lin AC Koltai PJ. Persistent pediatric obstructive sleep apnea and lingual tonsillectomy. Otolaryngol Head Neck Surg. (2009) 141:81–5. 10.1016/j.otohns.2009.03.011 19559963

[62] Thottam PJ Govil N Duvvuri U Mehta D. Transoral robotic surgery for sleep apnea in children: is it effective? Int. J. Pediatr. Otorhinolaryngol. (2015) 79:2234–37. 10.1016/j.ijporl.2015.10.010 26518466

[63] Villa MP Rizzoli A Miano S Malagola C. Efficacy of rapid maxillary expansion in children with obstructive sleep apnea syndrome: 36 months of follow-up. Sleep Breath. (2011) 15:179–84. 10.1007/s11325-011-0505-1 21437777

[64] Schwartz M Acosta L Hung Y-L Padilla M Enciso R. Effects of CPAP and mandibular advancement device treatment in obstructive sleep apnea patients: a systematic review and meta-analysis. Sleep Breath. (2017) 22:555–68. 10.1007/s11325-017-1590-6 29129030

[65] Sharples L Glover M Clutterbuck-James A Bennett M Jordan J Chadwick R. Clinical effectiveness and cost-effectiveness results from the randomised controlled trial of oral mandibular advancement devices for obstructive sleep apnoea-hypopnoea (TOMADO) and long-term economic analysis of oral devices and continuous positive airway pressure. Health Technol. Assess. (2014) 18:1–296. 10.3310/hta18670 25359435 PMC4781121

[66] Huynh NT Desplats E Almeida FR. Orthodontics treatments for managing obstructive sleep apnea syndrome in children: a systematic review and meta-analysis. Sleep Med. Rev. (2016) 25:84–94. 10.1016/j.smrv.2015.02.002 26164371

[67] Morovic CG Monasterio L. Distraction osteogenesis for obstructive apneas in patients with congenital craniofacial malformations. Plast. Reconstr. Surg. (2000) 105:2324–30. 10.1097/00006534-200006000-00003 10845284

[68] Verlinden CRA van de Vijfeijken SECM Jansma EP Becking AG Swennen GRJ. Complications of mandibular distraction osteogenesis for congenital deformities: a systematic review of the literature and proposal of a new classification for complications. Int. J. Oral Maxillofac. Surg. (2015) 44:37–43. 10.1016/j.ijom.2014.07.009 25148931

[69] Denny AD Talisman R Hanson PR Recinos RF. Mandibular distraction osteogenesis in very young patients to correct airway obstruction. Plast. Reconstr. Surg. (2001) 108:302–11. 10.1097/00006534-200108000-00004 11496167

[70] Tahiri Y Viezel-Mathieu A Aldekhayel S Lee J Gilardino M. The effectiveness of mandibular distraction in improving airway obstruction in the pediatric population. Plast. Reconstr. Surg. (2014) 133:352e−9e. 10.1097/01.prs.0000438049.29258.a8 24572880

[71] Stelnicki EJMD Lin W-YDDS Lee CDDS Grayson BHDDS McCarthy JGMD. Long-term outcome study of bilateral mandibular distraction: a comparison of treacher collins and nager syndromes to other types of micrognathia. Plast. Reconstr. Surg. (2002) 109:1819–25. 10.1097/00006534-200205000-00006 11994578

[72] Patel PA Shetye P Warren SM Grayson BH McCarthy JG. Five-year follow-up of midface distraction in growing children with syndromic craniosynostosis. Plast. Reconstr. Surg. (2017) 140:794e−803e. 10.1097/PRS.0000000000003879 28820838

[73] Meling TR Hans-Erik H Per S Due-Tonnessen BJ. Le Fort III distraction osteogenesis in syndromal craniosynostosis. J. Craniofac. Surg. (2006) 17:28–39. 10.1097/01.scs.0000194177.21916.f1 16432404

[74] Saltaji H Altalibi M Major MP Al-Nuaimi MH Tabbaa S Major PW Flores-Mir C. Le Fort III distraction osteogenesis versus conventional Le Fort III osteotomy in correction of syndromic midfacial hypoplasia: a systematic review. J. Oral Maxillofac. Surg. (2014) 72:959–72. 10.1016/j.joms.2013.09.039 24280172

[75] Arnaud E Di Rocco F. Faciocraniosynostosis: monobloc frontofacial osteotomy replacing the two-stage strategy? Childs Nerv. Syst. (2012) 28:1557–64. 10.1007/s00381-012-1853-2 22872273

[76] Collins B Powitzky R Robledo C Rose C Glade R. Airway management in pierre robin sequence: patterns of practice. Cleft Palate. Craniofac. J. (2014) 51:283–89. 10.1597/12-214 23875767

[77] Mahadevan M Barber C Salkeld L Douglas G Mills N. Pediatric tracheotomy: 17 year review. Int. J. Pediatr. Otorhinolaryngol. (2007) 71:1829–35. 10.1016/j.ijporl.2007.08.007 17953995

[78] Carr MM Poje CP Kingston L Kielma D Heard C. Complications in pediatric tracheostomies. Laryngoscope. (2001) 111:1925–8. 10.1097/00005537-200111000-00010 11801971

[79] Seligman KL Liming BJ Smith RJH. Pediatric tracheostomy decannulation: 11-year experience. Otolaryngol. Head Neck Surg. (2019) 161:499–506. 10.1177/0194599819842164 30987524

[80] Takahashi N Takano K Mitsuzawa H Kurose M Himi T. Factors associated with successful decannulation in pediatric tracheostomy patients. Acta Otolaryngol. (2017) 137:1104–9. 10.1080/00016489.2017.1326064 28504000

[81] Fauroux B Leboulanger N Roger G Denoyelle F Picard A Garabedian E-N. Noninvasive positive-pressure ventilation avoids recannulation and facilitates early weaning from tracheotomy in children. Pediatr. Crit. Care Med. (2010) 11:31–7. 10.1097/PCC.0b013e3181b80ab4 19752776

[82] Gerek M Binar M Physiology of hypoglossal nerve stimulation. Oper. Tech. Otolaryngol. Head. Neck Surg. (2015) 26:105–7. 10.1016/j.otot.2015.03.011

[83] Strollo PJ Soose RJ Maurer JT de Vries N Cornelius J Froymovich O. Upper-airway stimulation for obstructive sleep apnea. N. Engl. J. Med. (2014) 370:139–49. 10.1056/NEJMoa1308659 24401051

[84] Diercks GR Wentland C Keamy D Kinane TB Skotko B de Guzman V. Hypoglossal nerve stimulation in adolescents with down syndrome and obstructive sleep apnea. JAMA Otolaryngol. Head Neck Surg. (2018) 144:37–42. 10.1001/jamaoto.2017.1871 29098288 PMC5833588

[85] Caloway CL Diercks GR Keamy D de Guzman V Soose R. Update on hypoglossal nerve stimulation in children with down syndrome and obstructive sleep apnea. Laryngoscope. (2019) 130:E263–7. 10.1002/lary.28138 31219619

[86] Abel F Bajaj Y Wyatt M Wallis C. The successful use of the nasopharyngeal airway in Pierre Robin sequence: an 11-year experience. Arch. Dis. Child. (2012) 97:331–4. 10.1136/archdischild-2011-301134 22331679

[87] Glynn F Fitzgerald D Earley MJ Rowley H. Pierre Robin sequence: an institutional experience in the multidisciplinary management of airway, feeding and serous otitis media challenges. Int. J. Pediatr. Otorhinolaryngol. (2011) 75:1152–5. 10.1016/j.ijporl.2011.06.009 21764465

[88] Kochel J Meyer-Marcotty P Wirbelauer J Böhm H Kochel M Thomas W. Treatment modalities of infants with upper airway obstruction–review of the literature and presentation of novel orthopedic appliances. Cleft Palate. Craniofac. J. (2011) 48:44–55. 10.1597/08-273 20500074

[89] Wagener S Rayatt SS Tatman AJ Gornall P Slator R. Management of infants with Pierre Robin sequence. Cleft Palate. Craniofac. J. (2003) 40:180–5. 10.1597/1545-1569(2003)040&lt 12605525

[90] Leeies M Flynn E Turgeon AF Paunovic B Loewen H Rabbani R. High-flow oxygen via nasal cannulae in patients with acute hypoxemic respiratory failure: a systematic review and meta-analysis. Syst. Rev. (2017) 6:202. 10.1186/s13643-017-0593-5 29037221 PMC5644261

[91] Hutchings FA Hilliard TN Davis PJ. Heated humidified high-flow nasal cannula therapy in children. Arch. Dis. Child. (2015) 100:571–5. 10.1136/archdischild-2014-306590 25452315

[92] Hawkins S Huston S Campbell K Halbower A. High-flow, heated, humidified air via nasal cannula treats CPAP-intolerant children with obstructive sleep apnea. J. Clin. Sleep Med. (2017) 13:981–9. 10.5664/jcsm.6700 28728621 PMC5529135

[93] Joseph L Goldberg S Shitrit M Picard E. High-flow nasal cannula therapy for obstructive sleep apnea in children. J. Clin. Sleep. Med. (2015) 11:1007–10. 10.5664/jcsm.5014 26094930 PMC4543244

[94] Amaddeo A Khirani S Frapin A Teng T Griffon L Fauroux B. High-flow nasal cannula for children not compliant with continuous positive airway pressure. Sleep Med. (2019) 63:24–8. 10.1016/j.sleep.2019.05.012 31604152

[95] Overbergh C Installe S Boudewyns A Van Hoorenbeeck K Verhulst SL. The Optiflow^TM^ interface for chronic CPAP use in children. Sleep Med. (2018) 44:1–3. 10.1016/j.sleep.2017.11.1133 29530362

[96] Verhulst SL Franckx H Van Gaal L De Backer W Desager K. The effect of weight loss on sleep-disordered breathing in obese teenagers. Obesity. (2009) 17:1178–83. 10.1038/oby.2008.673 19265797

[97] Siegfried W Siegfried A Rabenbauer M Hebebrand J. Snoring and sleep apnea in obese adolescents: effect of long-term weight loss-rehabilitation. Sleep Breath. (1999) 3:83–8. 10.1007/s11325-999-0083-7 11898113

[98] Kalra M Inge T Garcia V Daniels S Lawson L Curti R. Obstructive sleep apnea in extremely overweight adolescents undergoing bariatric surgery. Obes. Res. (2005) 13:1175–9. 10.1038/oby.2005.139 16076986

[99] Taylor SJA Rennie K Jon C. Clinical outcomes of an inpatient pediatric obesity treatment program in the USA. Int. J. Adolesc. Med. Health. (2017) 31:20160141. 10.1515/ijamh-2016-0141 28598799

[100] Amaddeo A Frapin A Fauroux B. Long-term non-invasive ventilation in children. Lancet Respir. Med. (2016) 4:999–1008. 10.1016/S2213-2600(16)30151-5 27423917

[101] Caldarelli V Borel JC Khirani S Ramirez A Cutrera R Pépin J-L. Polygraphic respiratory events during sleep with noninvasive ventilation in children: description, prevalence, and clinical consequences. Intensive Care Med. (2013) 39:739–46. 10.1007/s00134-012-2806-7 23344829

[102] Fauroux B Lavis J-F Nicot F Picard A Boelle P-Y Clément A Vazquez M-P. Facial side effects during noninvasive positive pressure ventilation in children. Intensive Care Med. (2005) 31:965–9. 10.1007/s00134-005-2669-2 15924228

[103] Diep B Wang A Kun S McComb JG Shaul DB Shin CE. Diaphragm pacing without tracheostomy in congenital central hypoventilation syndrome patients. Respiration. (2015) 89:534–8. 10.1159/000381401 25924848

[104] Wang A Kun S Diep B Davidson Ward SL Keens TG Perez IA. Obstructive sleep apnea in patients with congenital central hypoventilation syndrome ventilated by diaphragm pacing without tracheostomy. J. Clin. Sleep Med. (2018) 14:261–4. 10.5664/jcsm.6948 29351818 PMC5786846

[105] Bach JR. The use of mechanical ventilation is appropriate in children with genetically proven spinal muscular atrophy type 1: the motion for. Paediatr. Respir. Rev. (2008) 9:45–50. 10.1016/j.prrv.2007.11.003 18280979

[106] Ryan MM. The use of invasive ventilation is appropriate in children with genetically proven spinal muscular atrophy type 1: the motion against. Paediatr. Respir. Rev. (2008) 9:51–4. 10.1016/j.prrv.2007.10.002 18280980

[107] Watters KF. Tracheostomy in infants and children. Respir. Care. (2017) 62:799–825. 10.4187/respcare.05366 28546379

[108] Fine-Goulden MR Ray S Brierley J. Decision making in long-term ventilation for children. Lancet Respir. Med. (2015) 3:745–6. 10.1016/S2213-2600(15)00377-X 26477553

